# Patient-reported outcomes in chronic GVHD: instruments, clinical utility, and survivorship integration

**DOI:** 10.1186/s41687-026-01080-7

**Published:** 2026-05-14

**Authors:** Xiaoli Liang, Shiqin Huang, Ruihao Huang, Jia Liu, Fengming Wang, Yao Quan, Lichao Liu, Lingyu Zeng, Yimei Feng, Xiaoqi Wang, Xi Zhang

**Affiliations:** 1 Xinqiao Hospital of Army Medical University, Chongqing, China; 2 Xuzhou Medical College, Xuzhou, China

**Keywords:** cGVHD, Allogeneic transplantation, PRO, Cancer survivors

## Abstract

Chronic graft-versus-host disease (cGVHD) is a leading driver of long-term morbidity after allogeneic transplantation. This review synthesizes evidence on patient-reported outcomes (PROs) across organ systems and survivorship domains, appraises psychometrics and minimal clinically important differences (MCIDs), and translates measures into routine care. Validated tools—including the Lee Symptom Scale (LSS), FACT-BMT, SF-36, PROMIS, and pediatric instruments—capture symptom burden and function beyond clinician ratings, and studies have demonstrated associations of some measures with survival, failure-free survival, and non-relapse mortality. Concordance between provider-rated and PRO-defined responses is limited, while PRO changes often track clinically meaningful improvement. We propose an implementation framework and two practical tables mapping measures to organ involvement and use-cases. Key barriers include workflow integration, patient burden, and cross-cultural adaptation-gaps that are pronounced in Chinese cohorts. Embedding PROs in trials and survivorship models can sharpen risk stratification, inform shared decisions, and improve patient-centered outcomes in cGVHD.

## Introduction

Allogeneic hematopoietic stem cell transplantation (allo-HSCT) is a curative therapy for hematologic malignancies, creating a growing population of long-term survivors [1–4]. However, chronic graft-versus-host disease (cGVHD) remains the most common late complication and a leading cause of non-relapse mortality [5]. Affecting 30–70% of recipients [6, 7], cGVHD involves multi-organ fibrosis and inflammation, leading to profound functional impairment and reduced quality of life (QoL) [8–10].

Patient-reported outcomes (PROs)—encompassing symptom burden, functional status, and health-related QoL—are crucial for capturing the patient experience, beyond clinician-rated measures [11]. Despite endorsements by regulatory bodies, the use of PRO measures (PROMs) in cGVHD care remains heterogeneous, hampered by a lack of standardization and cross-cultural validation, particularly in Chinese populations [12, 13].

This narrative review therefore: (i) compares and appraises core cGVHD-specific PROMs and their measurement properties; (ii) proposes a pragmatic framework for integrating PROs into routine clinical workflow; and (iii) identifies cross-cultural adaptation challenges with actionable solutions.

## Methods (literature search & selection)

This narrative review was based on a structured literature search of PubMed, Embase, and Web of Science for studies published from January 2005 to March 2026. Search terms included combinations of “chronic GVHD” with “patient-reported outcomes,” “PROMs,” “quality of life,” “Lee Symptom Scale,” and “PROMIS.” Eligible study types comprised validation studies of cGVHD-specific PRO instruments, prospective cGVHD cohort studies, pivotal therapeutic trials with predefined PRO endpoints, and systematic reviews of PRO measures in cGVHD. Both adult and pediatric cGVHD populations were considered. General HSCT survivorship studies were included only when they provided data directly relevant to cGVHD or reported cGVHD-specific subgroup findings. Case reports, editorials, conference abstracts without sufficient data, and studies focused exclusively on acute GVHD were excluded. Reference lists of key articles were also manually screened to identify additional relevant publications. Study selection and synthesis were performed narratively by the authors without duplicate screening or formal data extraction. As this article was designed as a narrative review rather than a systematic review, no formal risk-of-bias assessment or PRISMA-style study selection process was performed.

## Clinical evaluation

The severity of cGVHD is routinely assessed using the NIH global severity score, organ-specific scoring systems, or physician-assigned immunosuppression grades [14]. Dynamic reassessment is essential for guiding therapy [15, 16].

The NIH Consensus Conference established standardized organ scoring and overall response criteria in 2005, which were revised in 2014 to reflect emerging evidence [17, 18]. Although these validated measures capture clinically meaningful organ changes [19, 20], algorithms for defining global improvement or worsening show only modest agreement with physician assessments, particularly in mixed-response scenarios [21]. Moreover, NIH-defined objective responses correlate inconsistently with key clinical outcomes such as symptom burden, QoL, and survival [22].

In contrast, PROMs, particularly the Lee Symptom Scale (LSS), demonstrate superior prognostic value. Changes in PROs correlate significantly with failure-free survival (FFS), overall survival (OS), and non-relapse mortality (NRM) [23, 24]. PROs are helpful but cannot replace clinical evaluation in some cases. They cannot diagnose cGVHD or assign NIH severity scores—these require physical exams, organ tests, and biopsy when needed. Clinical decision-making also requires laboratory parameters, infection assessment, and treatment toxicity evaluation, which are not fully captured by PROs alone. Patients may not always report these correctly. Also, when some organs improve while others worsen, doctor-assessed organ scores are essential to understand complex responses that PROs may miss. Consequently, existing evidence strongly supports a dual assessment approach: integrating at least one cGVHD-specific PRO (e.g., LSS) with clinician-reported measures in both clinical trials and routine care. This dual approach facilitates more patient-centered treatment decisions, captures benefits not reflected in clinician scores, and aligns with frameworks such as the NMDP Patient-Centered Outcomes Research model [25]. This model emphasizes the physical, emotional, social, and financial domains of survivorship. To facilitate tool selection in both research and clinical settings, we summarize the core PROMs currently used in cGVHD (Table 1). The PROMs have unique characteristics and applicability. Among the available instruments, the LSS demonstrates the most robust responsiveness and cGVHD-specific validity, while PROMIS-29 offers superior feasibility with computerized adaptive testing (CAT) capability and T-score-based standardization. FACT-BMT and SF-36 provide valuable complementary information for long-term survivorship studies.

**Table 1:** Overview of PROM tools in cGVHD: type, domains, psychometric properties, MCID, and key trials

Tool	Type	Domains/Subscales	Recall Period	Score Range / Direction	Validity	Reliability	Responsiveness/ MCID	Feasibility	Key Trials
Lee Symptom Scale (LSS)	cGVHD-specific	Skin, Eye, Mouth, Lung, Nutrition, Energy, Emotional	7 days	0-100 (↑ worse)	Content: cognitive interviews; Construct: correlates with SF-36/FACT-BMT	α = 0.84–0.85; retest *r* = 0.79–0.81	5–6 points (7d version)[13, 26]	28–30 items, ~2 min	Ibrutinib [27–29], Ruxolitinib [27, 30], Belumosudil [27, 31], Axatilimab [27]
FACT-BMT	Transplant-specific	Physical, Social/Family, Emotional, Functional, BMT-specific	7 days	0-148 (↑ better)	Established for transplant QoL; limited cGVHD organ specificity	Not specifically reported for cGVHD	Varies (~ 5–7 points total) [32]	37 items, ~ 10–15 min	Multiple allo-HSCT survivorship studies
SF-36 / SF-12	Generic HRQoL	Physical & Mental Component Scores	4 weeks	0-100 (↑ better)	Generic; US normative data available	α > 0.80 across domains	0.3‑0.5 SD [33]	SF-12: 12 items, ~ 2 min	QoL cohorts, long-term follow-up
PROMIS-29	Generic PRO	Physical Function, Anxiety, Depression, Fatigue, Sleep, Pain, Social Role	7 days	T-score mean 50 ± 10	Construct: correlates with SF-36	α = 0.83–0.97 (Global Health)	0.5 SD (~ 5 points) [34]	29 items, ~ 5 min; CAT available	Ongoing HSCT registry studies
NIH Form B	cGVHD-specific clinician + patient parts	Organ-specific symptoms/function	Current	Varies by item	Aligned with NIH clinician scoring	Not routinely reported	NA	Clinician-administered	NIH consensus follow-up

The LSS continues to be the most popular cGVHD-specific tool, having been recommended by the NIH and included as a secondary endpoint in all four pivotal trials that resulted in FDA approval of cGVHD therapies [17, 27–31]. Its 7-day version has established MCIDs (5–6 points) [13, 26], making it appropriate for both clinical monitoring and trial endpoints; the FACT-BMT provides a more comprehensive assessment of transplant-related QoL, but lacks cGVHD-specific domains [32], which limits its sensitivity to organ-specific changes; the SF-36 and its abbreviated form, SF-12, are generic health-related quality of life (HRQoL) measures that allow comparisons with normative populations [34–36], but may miss cGVHD-specific symptoms. The PROMIS-29 provides efficient, standardized T-score-based assessment across multiple domains, with the advantage of computer-adaptive testing [34], but requires local normative data for interpretation [36]. The patient-reported components of the NIH Form B are directly aligned with clinician scoring [17], facilitating integrated assessment in specialized clinics.

As a result, the clinical or research setting should influence the instrument selection. We suggest the LSS as a fundamental cGVHD-specific instrument for routine cGVHD follow-up and interventional trials [17, 26], optionally augmented by a general QoL measure like PROMIS-29 or SF-12 to capture broader health consequences [34]. The SF-36 or FACT-BMT might be more suitable for survivorship studies that concentrate on long-term QoL [32, 36]. This multi-layered strategy strikes a compromise between patient burden, comprehensiveness, and specificity.

## Physical well-being

### Symptom

Longitudinal assessment post-HSCT identified tiredness, infection susceptibility, disinterest in sex, and weakness as the most common symptoms; notably, a high symptom burden at 4 months predicted delayed recovery at 1 year [37]. Lower self-efficacy was independently linked to greater symptom severity, poorer QoL, and higher distress [38]. In a multicenter survey, one-third of patients reported their most severe cGVHD symptoms persisting > 1 year, with dry eyes being the most bothersome (44%), substantially impairing activities of daily living such as eating, hygiene, and dressing [39]. In a separate study, symptom severity was found to increase with cGVHD grade, with severe disease associated with more pain, depression, and multi-organ involvement [40].

The Lee Symptom Scale (LSS), recommended by the NIH [17], has identified missing items (e.g., edema, vaginal, hepatic, nail symptoms) and minor ambiguities in the skin domain through cognitive interviews, though overall usability and patient acceptance were high [41]. The LSS has been validated in Latin American populations, showing good reliability and strong correlation with SF-36 and FACT-BMT [12]. A modified 7-day version improved internal consistency in the nutrition and lung subscales, and a 5–6 point change is considered clinically meaningful [26]. These features make LSS not only a trial endpoint but also a practical clinical follow-up tool.

### Quality of Life

QoL declines markedly in the early post-HSCT phase, with gradual improvement over time [42]. In this context, both acute and chronic GVHD reduce QoL [43], with multi-organ moderate disease or severe single-organ involvement particularly detrimental [11]. Beyond GVHD, other significant risk factors for poorer HRQoL include intensive conditioning regimens, female sex, younger age, low social support, and baseline psychological distress [44]. Chronic comorbidities, psychotropic medication use, and socioeconomic factors such as low income and employment disruption further contribute to the QoL burden [45]. Chinese data, though derived from non-validated translations of existing PRO instruments, indicate that haplo-cord HSCT may yield better physical and social functioning than MRD-HSCT, while moderate-severe cGVHD remains the strongest negative predictor [46].

For assessment, PROMIS tools show strong correlation with the SF-36 and serve as efficient alternatives for comprehensive assessment in cGVHD [34]. However, clinician-reported remission does not guarantee resolution of symptomatic burden, as issues like oral dryness, ocular discomfort, and poor nutrition commonly persist [47]. Organ involvement-especially skin sclerosis, joint/fascia restriction, and pulmonary disease-has the greatest adverse impact [48, 49]. Physician VAS ratings consistently overestimate patient well-being, especially in those with mild symptoms, underscoring the need for direct patient input [50].

### Disability

Cord blood and haploidentical HSCT recipients show significantly lower disability rates compared with mismatched unrelated PBSC transplants [51]. Disability was defined as NIH grade 2–3 keratoconjunctivitis sicca, scleroderma, joint/fascia involvement, grade 3 esophageal stricture, or any grade BOS [51]. This definition captures both functional limitations and irreversible organ damage, reflecting the multidimensional burden of cGVHD.

### Fatigue

Fatigue is common, unrelieved by rest, and linked to worse QoL [52]. It can persist for years, impairing function and cognition [53], with > 1/3 of cGVHD patients reporting clinically significant fatigue [54]. Predictors include reduced activity, pulmonary/musculoskeletal symptoms [54], older age, and hepatic/pulmonary involvement [55]. Furthermore, acute GVHD predicts fatigue at day 100, while cGVHD predicts fatigue at 1 year [56]. Combined models using NIH scores, LSS sleep/depression, and PG-SGA activity/function scores can identify high-risk patients [57].

### Physical function

cGVHD significantly impairs patients’ physical function and QoL, which is directly reflected in their reported increased symptom burden, limitations in daily activities, and diminished health perception [47]. The self-reported severity of symptoms is closely associated with declines in objective functional measures such as walking ability [58], while involvement of muscles/fascia and lungs [59, 60], as well as long-term glucocorticoid therapy, are key factors affecting patients’ functional experience [61]. Notably, functional gains parallel symptom improvement and are linked to better QoL [62]. Although objective function tends to be worse in older patients, their reported QoL may be comparable to that of younger patients, highlighting the subjective dimension of QoL assessment [63]. Importantly, these impairments are not irreversible. Targeted rehabilitation training [64] and specialized palliative care, along with other supportive interventions, can effectively improve patient-reported physical function, alleviate psychological distress, and thereby significantly enhance QoL [65, 66]. Therefore, in the management of cGVHD, comprehensive strategies aimed at symptom relief and functional improvement should be integrated to optimize the patient-reported experience.

### Cognitive function

Cognitive function, a critical dimension of long-term survivorship captured by PROMIS Cognitive Function and the LSS energy/psychological subscales, is increasingly recognized in cGVHD. Neurological manifestations of cGVHD encompass a broad spectrum of immune-mediated complications affecting both the peripheral and central nervous systems, including a range of peripheral and central nervous system conditions [67, 68]. Direct evidence from cGVHD populations confirms substantial cognitive burden. A large cross-sectional survey of patients with active cGVHD found that 47.0% reported severe cognitive disability affecting daily activities such as managing finances and social interactions [69]. In the broader HSCT survivorship literature, qualitative research has documented that survivors spontaneously report perceived neurocognitive decline after discharge, describing impacts on everyday tasks, work hour reductions, and early retirement [70]. Taken together, current evidence supports the inclusion of cognitive assessment in long-term cGVHD follow-up, although the cGVHD-specific literature remains limited. From an expert perspective, routine cognitive screening may be particularly valuable in patients with active multisystem disease, prolonged symptom burden, or significant functional complaints. These implications should be interpreted cautiously.

### Sexual function

Sexual health is a frequently under-assessed PRO domain in cGVHD care. This domain is included as it is captured by PRO instruments such as PROMIS Sexual Function and the FACT-BMT. Sexual dysfunction is common post-HSCT and linked to reduced QoL [71, 72]. Contributing factors include fatigue, poor health, cGVHD, recurrence anxiety, and body image concerns [73]. Moderate-severe GVHD increases ED risk sixfold [73]; male genital cGVHD occurs in 5–20% [74, 75] and is associated with hypogonadism [76]. Genital disease often coexists with other mucosal involvement [77]. Communication barriers limit diagnosis and management [78], highlighting the need for proactive screening, patient-provider dialogue, and multidisciplinary intervention. However, some of these implications are extrapolated from broader post-HSCT literature, and direct cGVHD-specific evidence remains limited.

In summary, evidence consistently shows that physical impairments in cGVHD—particularly fatigue, reduced mobility, and musculoskeletal or pulmonary involvement—are strongly linked to lower QoL and higher disability. Tools such as the LSS physical domains and PROMIS Physical Function efficiently capture these deficits, with MCIDs enabling sensitive monitoring. Routine integration of these instruments supports early rehabilitation and targeted supportive care.

## Emotional well-being

### Post-traumatic stress growth (PTSG)

HSCT is widely recognized as a potentially traumatic experience, and general HSCT survivorship studies document elevated rates of anxiety, depression, and post-traumatic stress disorder (PTSD) across the entire transplant population [79]. QoL declines are most pronounced during the initial hospitalization-regardless of conditioning intensity-and strongly predict subsequent PTSD symptoms [80]. Within this broader context, cGVHD imposes an additional and sustained psychological burden. Patients with active cGVHD report significantly higher distress levels than those without cGVHD [81]. Importantly, psychological distress and post-traumatic growth (PTG) may coexist. In general HSCT survivor cohorts, higher PTG correlates with female gender, younger age, receipt of psychosocial care, and better QoL [82]. Accordingly, whether active cGVHD facilitates or impedes PTG remains uncertain. In our view, social support is likely to remain clinically relevant in cGVHD, but this inference is based mainly on broader HSCT survivorship literature rather than direct cGVHD-specific evidence [83].

### Depression

Depressive symptoms are common after allo-HSCT. In broader HSCT survivorship studies, approximately 55% of recipients report depressive symptoms, and identified risk factors include younger age, female sex, impaired physical function, and ongoing immunosuppression [84]. Chronic pain and lower resilience have also been associated with worse mental health-related QoL in transplant survivors [85].

Importantly, in cGVHD cohorts specifically, depression is not merely a comorbidity but an independent predictor of adverse outcomes. The relationship between depression and cGVHD is considered bidirectional. Depression may result from cGVHD-related symptoms and functional impairment, but adherence to immunosuppressive therapy may be hampered by pre-existing depression or psychological distress prior to transplant, which could cause or worsen cGVHD [86]. This underscores the rationale for pre-transplant mental health screening and long-term PRO monitoring [87].

Critically, in cGVHD cohorts specifically, depression is directly linked to inferior clinical outcomes. A prospective study of patients with active cGVHD found that clinically significant psychological distress independently predicted reduced overall QoL, decreased physical and functional status, and worse overall survival [86]. Broader HSCT survivorship studies further demonstrate that pre-transplant psychological factors, such as negative illness perception and baseline distress, predict depression risk and poorer HRQoL at key post-HSCT time points [88].

### Anxiety

Anxiety is prevalent following HSCT. In the general HSCT survivorship setting, the INSPIRE study (NCT01602211) found that 32% of patients reported clinically significant anxiety [89]. The presence of cGVHD, lower income, and lack of internet access were independently associated with this distress [89]. Focusing specifically on cGVHD populations, among patients with moderate-to-severe cGVHD, anxiety symptoms were reported in 20–36% over 6 months [90]. Emotion-oriented coping, poor physical functioning, and higher symptom burden are key predictors of anxiety, whereas task-oriented coping appears protective [90]. These findings support anxiety as a relevant cGVHD outcome domain. More broadly, they also suggest that emotional well-being in cGVHD should be interpreted in close relation to symptom burden and functional limitation.

### Clinical intervention

This domain is included as psychosocial interventions are evaluated using PRO instruments such as PROMIS Depression/Anxiety and the LSS emotional subscale. Accordingly, the psychosocial effects of HSCT should be methodically addressed by clinical therapies. Positive psychological interventions have been shown to be possible [91]. Benefits last for up to six months, and integrated inpatient palliative care during hospitalization dramatically lowers the likelihood of post-transplant deterioration in QoL, symptom burden, and psychological distress [92]. For patients with cGVHD in particular, social support and self-efficacy can improve adherence and reduce distress [93].

Programs for multidisciplinary group support have produced encouraging outcomes. For moderate-to-severe cGVHD, the “Horizons Program” pilot showed promise in enhancing emotional health, symptom load, and QoL [94]. However, much of the interventional evidence derives from broader HSCT populations, and dedicated cGVHD-specific validation remains needed.

In summary, anxiety and depression are more common in cGVHD than in overall HSCT groups. Important factors include functional deterioration, symptom burden, and inadequate coping mechanisms. PROMIS Depression/Anxiety and the LSS emotional subscale are validated for clinical use, with established MCIDs that facilitate timely psychological or psychiatric referral. By implementing these strategies, proactive mental health support is made possible and may increase survival.

## Social well-being

### Economic burden

Financial toxicity is increasingly recognized as a PRO domain relevant to cGVHD survivorship. This domain is included as it is captured by PRO instruments such as the COST-FACIT and PROMIS economic burden items. cGVHD imposes substantial economic burdens, characterized by increased healthcare utilization, direct medical costs, and productivity loss. Steroid-refractory cGVHD entails particularly high resource use, with significantly more outpatient visits, hospital admissions, and overall healthcare expenditures within two years post-HSCT [95]. Real-world data confirm that moderate-to-severe cGVHD prolongs healthcare use and increases costs [96].

This financial strain correlates with adverse psychosocial outcomes. Over 66% of patients report significant financial burden, which is associated with higher rates of depression, anxiety, and sleep disturbances [97]. cGVHD also elevates the risk of financial hardship in younger survivors [96]. Notably, younger age, earlier disease onset, and greater psychological resilience predict better chances of returning to work or school [97].

### Return to work or study

This domain is included as return to work (RTW) represents a functional PRO endpoint captured by instruments such as PROMIS Social Roles and SF-36 role functioning domains. RTW is a critical milestone in post-HSCT rehabilitation [98]. General HSCT cohort studies indicate that recovery is frequently prolonged: 76% of patients remain on medical leave one year following allo-HSCT [99]. This has a major effect on QoL, which is directly related to economic security and job stability [97, 100]. The broader survival implications of socioeconomic determinants are shown by the independent association of a lower socioeconomic level with greater risks of both NRM and all-cause death [101]. 

cGVHD substantially exacerbates RTW barriers. Direct evidence from cGVHD-specific surveys shows that 61.3% of patients with cGVHD take disability leave, 33.8% quit employment entirely, and 71.3% report income loss attributable directly to cGVHD [102]. The 3-year incidence of cGVHD-related impairment can surpass 55% in specific transplant circumstances (e.g., HLA-mismatched peripheral blood), significantly restricting RTW rates [103]. Crucially, not going back to work is a predictor of worse OS and a higher load of symptoms [104].

### Social function

Social functioning reflects a patient’s ability to participate in interpersonal relationships, social activities, and community roles. In general HSCT survivorship studies of adolescent and young adult recipients, the strongest predictors of impaired social functioning were fatigue, distress, and physical limitations. Active cGVHD accounted for only 5.5% of variance in this broad cohort, whereas fatigue and physical function accounted for 46.6% and distress and social support for 7.7% [105].

In contrast, direct comparisons within cGVHD populations reveal more pronounced deficits. QoL studies using the QLQ-C30 and SF-36 demonstrate that patients with active cGVHD consistently report significantly poorer social functioning scores than those without cGVHD. Differences are particularly notable in the QLQ-C30 social functioning domain and the SF-36 physical functioning domain [35]. These findings highlight the importance of validated PROMs that capture social dimensions of survivorship in routine cGVHD evaluation.

In summary, cGVHD often disrupts employment, financial stability, and social reintegration. Social role impairment is under-recognized clinically but contributes significantly to distress and poor long-term outcomes. PROMIS Social Roles and SF-36 Social Functioning domains offer efficient monitoring, while integration into survivorship clinics can guide vocational rehabilitation and social support interventions.

## Organ-specific assessment

### Skin

The NIH skin score remains the reference standard for cutaneous cGVHD assessment [17, 18]. Cutaneous cGVHD comprises heterogeneous epidermal and sclerotic manifestations [17]; to better capture the distinct burden of sclerosis, the Lee Symptom Scale–Skin Sclerosis (LSS-SSc) was recently developed and validated as a cGVHD-specific PRO tool [106]. Erythematous Body Surface Area (BSA) involvement is prognostically relevant, with each 10% increase linked to a 33% higher NRM risk and 28% higher mortality [107]. In patients with skin of color, erythematous or sclerotic changes were associated with worse clinician severity scores, lower FACT-BMT, and higher LSS scores, with notable physician-patient discordance even in inactive disease [108]. FACT-BMT and LSS skin subscale deteriorations-7 and 11 points respectively-were associated with 9–16% increased mortality per worsening unit [32]. Patients with the lowest scores had the poorest OS and highest NRM. These data underscore the need to integrate PROs with dermatologic scoring to improve prognostic accuracy and therapeutic decision-making.

### Ocular involvement

The ICCGVHD score ≥ 6 (with systemic GVHD) or ≥ 8 (without) defines definite oGVHD and correlates strongly with NIH ocular scores [109, 110]. oGVHD is associated with significantly poorer QoL, cognitive impairment, and is influenced by disease severity, economic burden, and visual demands [111, 112]. Objective signs such as corneal staining, Schirmer’s test, and tear film breakup time are linked to lower vision-related QoL [113, 114]. Longitudinal studies show that OSDI, SANDE, and corneal staining improve with treatment, whereas Schirmer’s declines over time, suggesting that symptom scores and corneal staining are more sensitive to change than tear production [115]. The Lee Eye Subscale, with good sensitivity and specificity, is a promising PRO for detecting active oGVHD [116].

### Oral involvement

Oral cGVHD leads to salivary gland dysfunction, mucosal erythema, lichenoid changes, and ulcerations, resulting in pain, sensitivity, and functional impairment [117]. The NIH-GSS and Lee Symptom Scale oral items capture these impacts, while the modified OMRS provides objective scoring (0–12, ≥ 2 clinically significant, ± 2 indicating change) [17, 118]. In studies using NIH-GSS and OHIP-14, OHRQoL was most negatively affected by pain and sensitivity, with only weak correlation to lesion severity [119, 120]. Xerostomia prevalence ranges from 43 to 77% post-transplant [120, 121]. Given the inconsistent link between lesion severity and QoL, symptom-focused PROs are critical in oral cGVHD evaluation.

### Joints and fascia

A 2-point change in PROM is considered clinically meaningful, while a change from 0 to 1 on the NIH joint/fascia score does not necessarily indicate worsening [122]. Using both NIH and PROM scores improves detection of improvement and deterioration [123].

### Pulmonary involvement

In a prospective study, patients developing BOS after allo-HSCT had persistently worse HRQoL scores on QLQ-C30 and SF-36 PCS, independent of other cGVHD severity [124]. (Table 2)

**Table 2:** Organ-specific monitoring in chronic GVHD: clinical measures, patient-reported outcomes, and clinical interpretation

Organ/System	Clinician Measures	Recommended PRO/PROM	Interpretation
Skin	NIH Skin Score, Body Surface Area (BSA)%	LSS-Skin Sclerosis (LSS-SSc)	BSA increase ≥ 10% indicates elevated non-relapse mortality (NRM) risk [107]; Worsening skin PRO reflects increased symptom burden [32]
Eye	ICCGVHD score, Corneal staining	OSDI, SANDE	OSDI improvement reflects function change more than Schirmer’s [115]
Oral	NIH Oral Mucosa Rating Scale (NIH OMRS)	LSS-Mouth, OHIP-14	OMRS ≥ 2 indicates moderate-severe mucosal involvement requiring intensified intervention [125]
Lung	Pulmonary Function Tests (PFTs), NIH Lung Score	LSS-Lung	FEV₁ decline ≥ 10% or PRO worsening suggests disease activity/progression, requiring exclusion of infection [124]
Joint/Fascia	Range of Motion (ROM) measurement	LSS-Joint	ROM limitation > 20° with PRO worsening indicates active fascial involvement causing functional impairment [122, 123]

## Special populations

Pediatric and older adult survivors present distinct challenges for PRO assessment and interpretation. In children, the use of proxy reports and age-adapted tools (e.g., PedsQL) is necessary, and active cGVHD remains a primary driver of impaired HRQoL [126]. For older adults, comorbidities, functional status, and polypharmacy must be considered, as they significantly influence outcomes and PRO scores [127]. In both populations, standard PRO instruments may require contextualization. Geriatric assessment (GA) offers a validated framework for older patients to interpret PROs in the context of their general health and guide treatment decisions [128]. The inclusion of core assessments like comorbidity indices (like HCT-CI), functional measures (like gait speed and grip strength), and cognitive screening is supported by growing evidence, even though recommendations regarding specific GA components are still changing. These assessments have shown prognostic relevance in older HSCT recipients [129, 130]. Routine GA use is still hampered in many centers by implementation hurdles like staff training gaps and time constraints [131]. Ultimately, a tailored approach to PRO strategy—informed by age-appropriate assessments—is essential to ensure accurate and meaningful patient-centered care across the age spectrum.

## Cross-cultural adaptation challenges and solutions

Globally, there are substantial cross-cultural obstacles to the extensive clinical application of PROs in cGVHD, despite their increasing acknowledgment. To guarantee content validity and comparability across various linguistic and cultural contexts, strict translation and cultural adaptation are necessary in accordance with ISPOR criteria [12, 132]. But few centers use PROs regularly. Problems include staff training, workflow, and no local data [13, 36, 133, 134]. Many languages lack validated tools like LSS and PROMIS-29 [12, 13]. There are no local norms for scores [36]. Culture also affects how patients report symptoms—some may not talk about distress or sex issues. For example, studies have demonstrated the impact of cGVHD on QoL [46], yet these investigations often used ad hoc translations rather than fully validated tools—a situation mirrored in many other non-Western populations. To address these universal challenges, we propose a multi-faceted approach: conducting multicenter international validation studies following ISPOR methodology [132]; establishing population-specific normative data through large-scale surveys across diverse geographic regions [36]; deriving local MCIDs; and integrating culturally adapted ePRO platforms into routine care and multicenter registries [20, 134]. Such efforts are essential to enable accurate PRO assessment across diverse populations, facilitate meaningful international comparisons in clinical trials, and ultimately ensure that patient-centered outcomes are validly measured in all cGVHD survivors worldwide.

To assist readers in interpreting the strength of evidence across the topics covered in this review, Table 3 provides a structured overview that distinguishes cGVHD-specific data, broader HSCT survivorship findings, and expert interpretation. This table is based on the body of evidence reviewed in the preceding sections. Evidence levels were defined as follows: Extensive, multiple prospective cGVHD cohort studies or PRO validation studies available; Moderate, at least one cGVHD-specific study or multiple studies with cGVHD subgroup analyses; Limited/Minimal, conclusions rely largely on general HSCT survivorship data; Supporting, general HSCT data provide complementary evidence; Expert interpretation, drawn from clinical consensus.

**Table 3:** Level of evidence across survivorship domains reviewed

Domain	cGVHD-Specific Evidence	HSCT Survivorship Findings	Expert Interpretation
Symptom burden	Extensive	Supporting	-
Quality of life	Extensive	Supporting	-
Fatigue	Moderate	Supporting	-
Physical function	Moderate	Supporting	-
Cognitive function	Limited	Moderate	Screening recommendation
Sexual function	Limited	Moderate	Screening recommendation
Depression / Anxiety	Moderate	Extensive	-
Post-traumatic growth	Minimal	Moderate	Social support hypothesis
Economic burden	Moderate	Supporting	-
Return to work	Extensive	Supporting	-
Social function	Moderate	Moderate	-

## Discussion

Drawing on the evidence synthesized in this review, we propose an expert-informed, three-tiered core PRO set for consideration in cGVHD management. This framework is intended as a hypothesis-generating model to guide future implementation research, rather than an established clinical standard.

The proposed set comprises: (i) a cGVHD-specific tool, the LSS, preferably the 7-day version with established MCIDs of 5–6 points, to measure organ-specific symptom burden [13, 26]; (ii) a generic QoL measure, either SF-12 for its brevity and widespread use, or PROMIS-29 for its effectiveness and T-score-based standardization, to evaluate broader physical and mental health impacts [34, 36]; and (iii) a single-item fatigue screen (e.g., PROMIS Fatigue or SF-36 Vitality scale item), given the high prevalence and prognostic significance of fatigue in cGVHD [54]. This core set strikes a balance between patient burden, comprehensiveness, and specificity, needing around 10 to 15 min. When clinically indicated, organ-specific modules (such as LSS-SSc for skin sclerosis, OSDI for ocular involvement) or domain-specific tools (such as PHQ-4 for anxiety-depression, or PROMIS Social Roles for social functioning) can be added to this core for clinical trials or specialty clinics [86, 90, 106, 135].

**Fig. 1:**
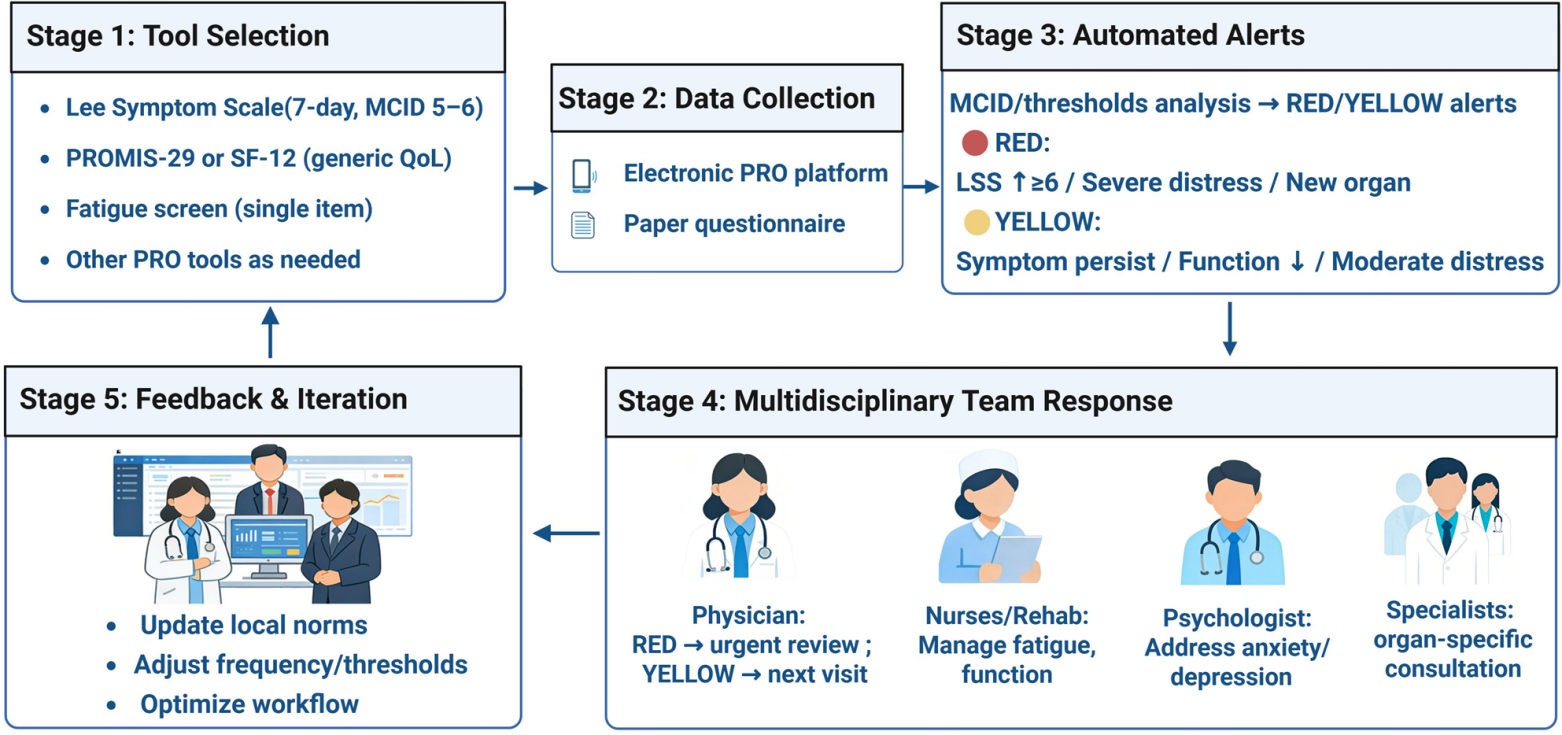
Proposed conceptual framework for integrating patient-reported outcomes in cGVHD clinical workflows

To operationalize this approach conceptually, we offer a five-stage framework for consideration (Fig. 1). This model has not been prospectively validated and should be viewed as an expert-informed proposal requiring iterative refinement based on local feasibility and emerging evidence. First, patients fill out core PROs before each visit (online or paper) (Stage 1–2). Second, data go into the electronic health record and are checked against MCID-based cutoffs. The system could be used to generate red flags (LSS increase ≥ 6 points, severe distress, new organ involvement) indicating need for urgent clinical review, and yellow flags (persistent symptom worsening, functional decline, moderate distress) signaling need for close monitoring (Stage 3). Third, a layered multidisciplinary team responds based on alert levels: physicians may help prioritize red flag cases for 24-hour review; nurses and rehabilitation therapists address fatigue and functional impairment; psychologists provide support for elevated distress; and specialists are consulted for organ-specific issues as detailed in Table 2 (Stage 4). Finally, accumulated data enable iterative refinement of local norms, alert thresholds, and workflow efficiency, addressing cross-cultural adaptation needs that remain particularly pressing in populations where validated PRO translations and normative data are lacking (e.g., Chinese cohorts) (Stage 5).

Professional teams are empowered to monitor QoL trajectories thanks to this tiered approach, which also allows for standardized measurement at critical timepoints and initiates multidisciplinary interventions when predetermined thresholds are crossed. Importantly, these relationships are bidirectional—physical symptoms may precipitate psychological distress, while pre-existing psychosocial vulnerabilities can increase cGVHD risk, underscoring the need for longitudinal PRO assessment beginning pre-transplant.

The NIH’s support for PROs and their inclusion as secondary or exploratory endpoints in the four pivotal trials for FDA-approved cGVHD treatments is supported by the data compiled in this review [27–31]. This paradigm shift toward “patient-defined recovery” is further solidified by the recent ASTCT consensus [135]. Although we draw attention to the Chinese context, these implementation and validation issues are present in all healthcare systems. In order to guarantee that the patient’s voice becomes the real compass directing cGVHD care globally, it is essential to develop localized instruments, establish population-specific norms, and establish shared decision-making platforms.

## Data Availability

No datasets were generated or analysed during the current study.

## Abbreviations

cGVHD: chronic graft-versus-host disease
PROs: Patient-reported outcomes
MCIDs: Minimal clinically important differences
LSS: The Lee Symptom Scale
Allo-HSCT: Allogeneic hematopoietic stem cell transplantation
QoL: Quality of life
PROMs: Patient-reported outcome measures
PROMIS: Patient-Reported Outcomes Measurement Information System
FFS: Failure-free survival
OS: Overall survival
NRM: Non-relapse mortality
HRQoL: Health-related quality of life
PTSG: Post-traumatic stress growth
PTSD: Post-traumatic stress disorder
PTG: Post-traumatic growth
RTW: Returning to work
OSDI: Ocular surface disease index
BSA: Body surface area
COST: Comprehensive Score for Financial Toxicity
CAT: Computerized Adaptive Testing
ED: Erectile Dysfunction
